# Hybrid Detection of Breast Abnormalities Based on Contrast Agents: Introducing a Proof of Concept from a Physics Perspective

**DOI:** 10.3390/s22197514

**Published:** 2022-10-03

**Authors:** Panagiotis Liaparinos

**Affiliations:** Radiation Physics, Materials Technology and Biomedical Imaging Laboratory, Department of Biomedical Engineering, University of West Attica, Ag. Spyridonos, 12243 Athens, Greece; liapkin@uniwa.gr

**Keywords:** breast imaging, contrast agents, hybrid detectors

## Abstract

This manuscript attempts to present a proof of concept from a physics perspective of a hybrid detective system based on the utilization of contrast agents with the purpose of indicating breast tissue abnormalities. In the present concept, the photon-counting module of the detector is set up to the K-characteristic radiation emitted by the contrast agent. Τwo X-ray spectra were used: 40 kV- W/Al (1.6 mm) and 50 kV- W/Al (1.6 mm) with additional filtration of 0.3 mm Gd. Iodine (I) contrast agent was studied as a ‘‘fingerprint’’ for tissue abnormality indication. A computational Monte Carlo model, based on previously published validated tabulated data and tissue experimental measurements, was developed with the purpose of showing that the present concept has practical potential; however, with a weakness of not being accompanied by experimental validation. The study considered two types of internal tissue layers (fibrous/tumor with thickness values of 0.2–2.5 mm) within an external layer of fat tissue (4 and 8 cm). Quantitative (number of encountered *K*-photons) and qualitative (tumor–fibrous ratio) advantages of using X-ray spectra of a higher tube voltage (50 kV) and of counting the *Κ_α_* photons were found. In addition, the quantitative and qualitative benefits were correspondingly more dominant at high (2.5 mm) and low (0.2 mm) tissue thickness values. In conclusion, by utilizing suitable contrast agents as ‘‘fingerprint’’ tissue abnormalities, the acquisition of combined morphological and functional imaging features (through the counting of *K*-X-rays) could enhance breast imaging in its present form and lead to advanced prognostic capabilities of breast abnormalities.

## 1. Introduction

The significance of medical imaging for human pathology investigations has largely been recognized within the Health Sciences community. Imaging science and technology are able to show and combine morphological, functional, and metabolic information [1]. During the staging of cancer (i.e., from screening and biopsy guidance for detection up to therapy response and palliation), multiple biomedical imaging techniques are used. A large portion of methods and systems for medical imaging (either in vivo or vitro) were developed on the basis of accurate detection of ionizing and non-ionizing radiation transmitted through or emitted by the human body [2]. However, although cancer research currently shows great progress, prognostic imaging validity still can be considered a Pandora’s Box. Over the past few decades, several spectacular innovations have been introduced and significant medical imaging modalities have been developed and later applied in clinical routine (e.g., mammography, ultrasound, radionuclide imaging, magnetic resonance imaging, optical imaging, etc.). Additional diagnostic tools (e.g., in vitro tissue analysis) have also assisted clinical decision-making. However, the development of hybrid imaging modalities, beyond the prominent clinical example of PET/CT and the emerging PET/MRI scans, is lately considered a common practice for overall imaging optimization [3,4].

Breast cancer is considered one of the most common health concerns and, in 2018, an estimation of more than 2 million new cases have annually been counted, approximately 25% of all new cancer cases in women [5]. In addition, after breast cancer metastasis, the 5-year survival rate drops dramatically, which is an additional reason to focus on early detection [6]. Under the circumstances of early detection, the subsequent treatment is more effective and a cure is more likely (a reduction of the risk of dying from breast cancer by 15% to 20%). X-ray mammography is thought as the major screening method for breast cancer diagnosis since this type of examination reduces mortality from breast cancer [7,8]. The efficacy of X-ray mammography depends upon the ability to identify cancers based on the differing absorption of X-rays in cancerous tissue compared to adipose and glandular tissue and depends on several issues, e.g., family history of cancer, woman’s age, index of body mass, the use of computer assisting tools, etc. [9]. However, a point worth commenting on is that not all breast cancers are detectible by the conventional form of the mammogram, the benefits for younger women are unclear [10,11,12], and the sensitivity of X-ray mammography decreases in women with dense breasts [13] who may present a higher risk of breast cancer [14], or on the other hand, most women who are recalled for further examination, perhaps including biopsy after a suspicious mammogram, do not have cancer. There are other imaging configurations, mainly based on non-ionizing radiation, such as Ultrasound (US) and Magnetic Resonance Imaging (MRI), which seem to be functional in specific cases, while they seem inappropriate for the cases of asymptomatic women [10,15]. These configurations are often combined with the traditional X-ray mammography but they are characterized by limitations, including the high cost, the limited specificity (MRI) or the low sensitivity (Ultrasound) [9]. Regarding functional imaging, positron emission tomography (PET) is usually employed for the examination of metastatic status, although there are common difficulties that arise from the use of radioactive isotope injection [9]. Other non-ionizing modalities, such as Optical Imaging (OI), could also help in the detection of pathophysiological features (e.g., blood flow/concentration) by assessing the optical properties of tissues and by helping spectroscopically improve the specificity of a suspicious lesion seen in X-ray mammography [10].

In addition, certain imaging techniques were developed with the purpose of indicating suspicious regions of possible pathological findings by either exploiting contrast agents or using different energy regions of the beam. Amongst them, the most important are: breast tomosynthesis and contrast-enhanced mammography (temporal or spectral) [16]. While existing imaging modalities and techniques have significantly helped the administration of breast cancer, no single diagnostic technique is appropriate for the multiple problems that arise in clinical practice. Therefore, new advanced studies in breast cancer diagnosis would strengthen the current technological achievements. The present article attempts to present a proof of concept from a physics perspective of a hybrid detective system by counting the K-characteristic radiation emitted by the contrast agent. A computational Monte Carlo model, based on previously published validated tabulated data and tissue experimental measurements (analytically presented in Section II.C), was developed with the purpose of showing the proof of concept; however, the completion of the idea needs experimental tests in the future.

## 2. Materials and Methods

### 2.1. The Hybrid Detective Imaging System

**Figure 1:**
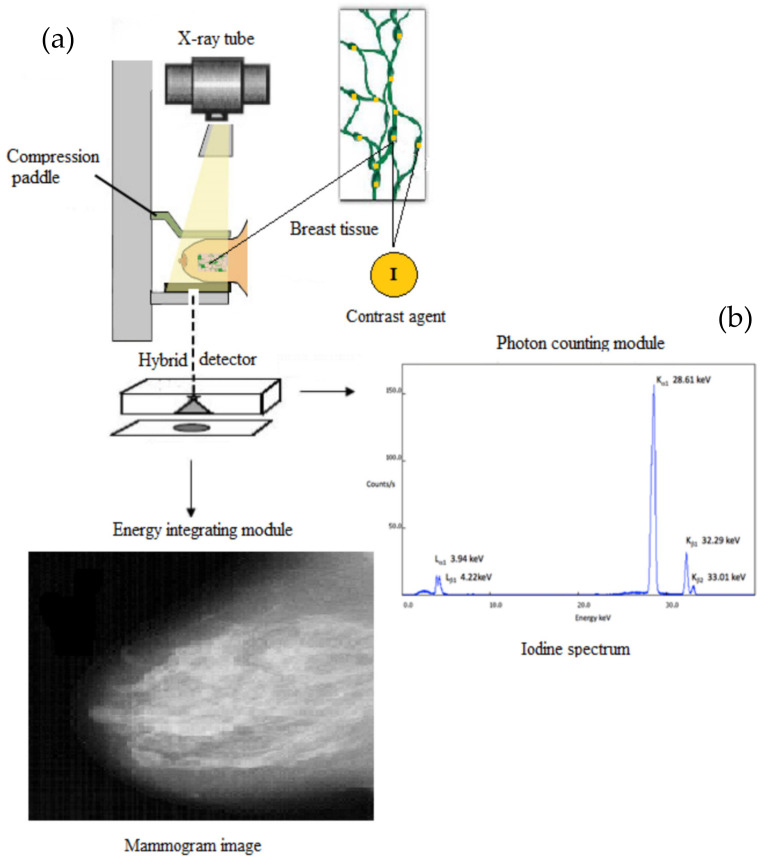
The hybrid detective system consists of: (**a**) the energy integrating module with the purpose of obtaining and characterizing the morphological imaging features; and (**b**) the photon-counting module with the purpose of obtaining and characterizing the functional imaging features by counting the K-characteristic radiation emitted by the contrast agent (the spectrum of the iodine contrast agent is illustrated).

The detective system, based on a hybrid detector, consists of: (a) the energy integrating module with the purpose of obtaining and characterizing the morphological imaging features; and (b) the photon-counting module with the purpose of obtaining and characterizing the functional imaging features. The design of such a hybrid detective system is illustrated in Figure 1. An X-ray beam is produced from an X-ray tube, X-rays impinge breast tissue, a fraction passes and the outcoming signal is recorded by a dual-function detector. In particular, the photon-counting module is based on the K-characteristic radiation emitted by the utilization of a suitable contrast agent.

### 2.2. The Role of the Contrast Agent as “Fingerprint” Tissue Abnormality

**Figure 2:**
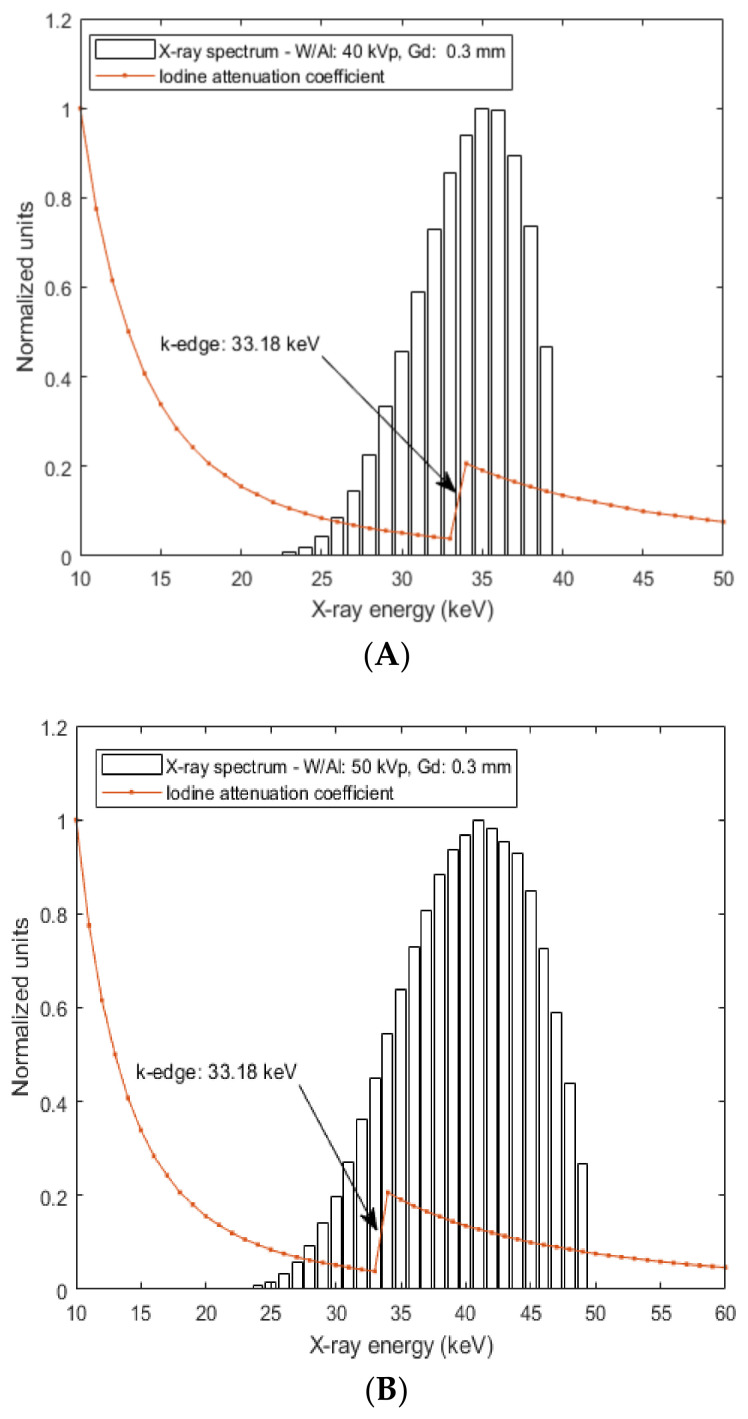
Figure illustrates two X-ray spectra: (**A**) 40 kV- W/Al (1.6 mm) and (**B**) 50 kV- W/Al (1.6 mm) with additional filtration 0.3 mm Gd. The simulation of the X-ray spectrum was carried out by an X-ray simulation tool [17]. In addition, the variation of the attenuation coefficient as a function of the X-ray energy (k-edge 33.18 keV) is also provided for the Iodine (I) element. The calculation was carried out using XmuDat database [18].

One of the largest problems associated with the utilization of contrast agents in hybrid systems is the lack of appropriate properties corresponding to multiple medical diagnostic tasks. In the proposed system, the choice of contrast agent as ‘‘fingerprint’’ tissue abnormality under qualitative and quantitative data analysis can be based on the following considerations: (a) in the energy integrating module, the characterization of the morphological imaging features is related to different X-ray attenuation profiles based on the attenuation properties of the X-ray beam. In this case, contrast enhancement can be achieved due to the suitable value of iodine k-edge (33.18 keV) for the X-ray tube voltages used in the present study (40–50 kV). Figure 2 shows that for the two X-ray spectra (40 kV- W/Al (1.6 mm) and 50 kV- W/Al (1.6 mm) with additional filtration 0.3 mm Gd), contrast enhancement can be achieved due to the suitable value of the iodine k-edge (33.18 keV) since high fraction of X-ray photons can be absorbed and (b) in the photon-counting module the characterization of the functional imaging features can be related to different attenuation profiles of *K*-X-rays produced (within the breast tissue configuration) and emitted (out of the breast tissue configuration) by the iodine contrast agent (Κα: 28.46 keV and Κβ1: 33.29 keV). This issue is modeled and analyzed through Monte Carlo simulation in the following subsection.

### 2.3. Monte Carlo Modeling of K-X-rays Emitted by the Contrast Agent

**Figure 3:**
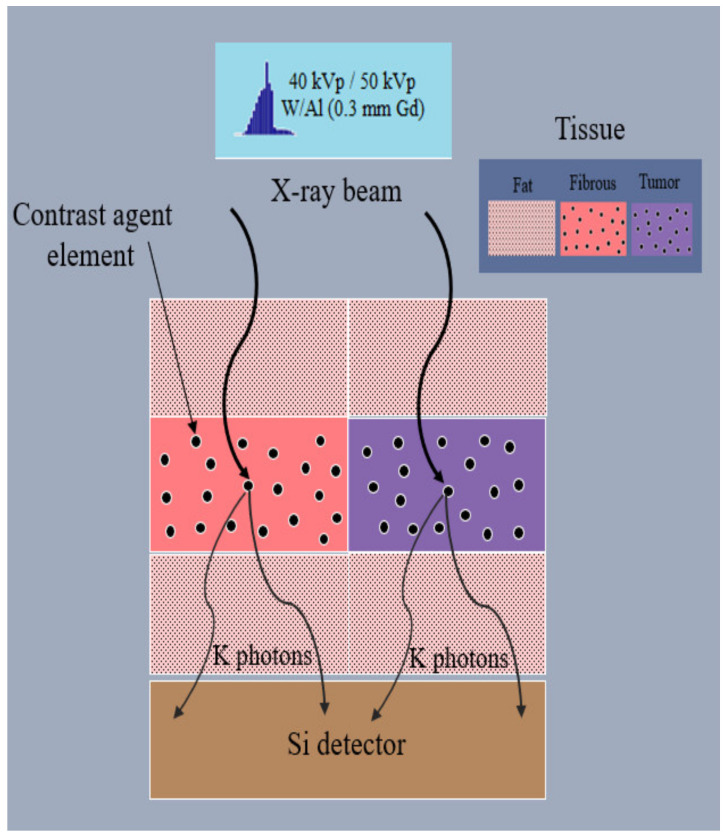
Figure illustrates the geometry of the model. An X-ray beam (a number of X-ray photons sampled by an X-ray spectrum corresponding to 10 mGy or 15 mGy ESAK) impinges upon two different configurations of breast tissue (external fat tissue and internal fibrous/tumor incorporating a fraction of contrast agent elements) and thereafter, by interacting through photoelectric absorption, *K*-X-rays are produced, emitted and finally detected by a Si converter layer.

The geometry of the computational Monte Carlo model is illustrated in Figure 3.

Two different configurations of breast tissue were considered. Each configuration contains an internal tissue layer (either fibrous or tumor) incorporating uniformly distributed atoms of different concentrations (i.e., number of contrast agent elements per unit tissue mass). An X-ray photon history is considered to start when an X-ray photon with energy sampled from an X-ray spectral distribution impinges upon the mass of the tissue. The incident X-ray energy fluence, *Ψ_x_*, is expressed by the following equation:

(1) $$\Psi_{x}(E)=\int_{0}^{E_{0}}N_{X}(E)EdE$$

where *Eo* is the maximum spectral energy and (*E*) is the X-ray energy spectrum, i.e., the number of the X-ray photons N_X_ with corresponding X-ray energy *E* at the interval (0, *Eo*]. Two X-ray spectra were used in the present study: 40 kV- W/Al (1.6 mm) and 50 kV- W/Al (1.6 mm) with additional filtration of 0.3 mm Gd. The simulation of the X-ray spectra was carried out by the X-ray simulation tool taken from Punnoose et al. [17]. The X-ray photon transport follows a multi-layer tissue consisting of three sub-layers: (i) an external upper layer *d*_1_ of fat tissue (with thickness 2 cm or 4 cm); (ii) an internal layer *d*_2_ of either fibrous or tumor tissue of thickness between 0.2 mm and 2.5 mm, incorporating contrast agent elements; and (iii) an external lower layer *d*_3_ of fat tissue (with thickness 2 cm or 4 cm). The total thickness of the fat tissue layer corresponds to 4 cm or 8 cm which corresponds to average and large (highly dense) sized breasts, respectively. The initial step length *S*_1_ is obtained as follows:

(2) $$S_{1}=-\frac{1}{{\left(\mu_{att}\right)}_{Fat}(E)}\ln R_{1}$$

where *R*_1_ is a random number uniformly distributed in the interval (0, 1] and (*μ_aIFat_*(*E*) is the linear attenuation coefficient of the fat tissue. Thereafter, if *S*_1_ > *d*_1_, the X-ray photon history continues in the second layer traveling the remaining path length–*S*_2_ = *S*_1_ − *d*_1_, which is converted for the new tissue, as given below:

(3) $$S_{2}\prime =S_{2}\frac{{\left(\mu_{att}\right)}_{Fat}(E)}{{\left(\mu_{att}\right)}_{CA}(E)\;d_{CA}\;pd_{CA}}$$

where I*_att_*)*_CA_*(*E*) is the linear attenuation coefficient of the contrast agent (iodine) and *d_CA_* is the density (i.e., the concentration) of the contrast agent elements within the tissue layer. Typical values of iodine contrast agent concentration [19] are 300 mg/mL and 370 mg/mL. In this study, *d_CA_* was assumed to be 300 mg/mL. In Equation (3), *pd_CA_* is the packing density of the contrast agent elements within the tissue layer which differs between normal and tumor tissues. In the present study, the values of packing density were chosen based on the so-called “volume of tracer distribution” which corresponds to the volume of water in tissue that exchanges with a unit volume of water in arterial blood and depends on the tissue composition. These values for breast were assumed to be 0.14 for normal tissue and 0.56 for tumor [20], corresponding to actual concentrations in tissues 42 mg/mL and 168 mg/mL, respectively.

Ιf *S*_2_′ < *d*_2_, at the site of the X-ray photon interaction, the X-ray photon is assumed to interact considering only the X-ray photons above the binding energy of the contrast agent element. Setting a random number *R*_2_, uniformly distributed in the interval (0, 1], the X-ray photon is assumed to undergo photoelectric absorption if *€*≤ (*p_pe_*)*_CA_*(*E*), and thereafter a sequence of processes may occur depending on its energy *E*. The probability of photoelectric effect occ€ence (*p_pe_*)*_CA_*(*E*) is determined by the relative probability of photoelectric effect with respect to the scattering events (Rayleigh and Compton) as given below:

(4) $${\left(p_{pe}\right)}_{CA}(E)=\frac{{\left(\mu_{pe}\right)}_{CA}(E)}{{\left(\mu_{att}\right)}_{CA}(E)}$$

where (*μ_pe_*)*_CA_*(*E*) is the partial interaction coefficient of photoelectric effect taken from XmuDat [18]. The photoelectric absorption may occur either in the K-shell or in the L-shell. The probability of photoelectric absorption in the K-shell is obtained as follows:

(5) $$p_{K}(E)=\frac{\sigma_{pe}^{K}(E)}{\sigma_{pe}^{K}(E)+\sigma_{pe}^{L}(E)}$$

where $\sigma_{pe}^{K}$ and $\;\sigma_{pe}^{L}$ are the corresponding probabilities of K-shell and L-shell (the summation of L-subshells, i.e., *L*_1_, *L*_2_ and *L*_3_ subshells) contributions to the photoelectric effect. Numerical values were taken from EPDL97 data library [21]. Setting a random number *R*_3_, photoelectric absorption occurs in K-shell if *R*_3_ ≤ *p_K_*(*E*) and thereafter K-photons are emitted. The choice of either *Kα* photons emission (due to *KL*_1_ relaxation) or *Kβ* photons emission (due to *KM*_1_ relaxation) is based on the probability of *KL* relaxation *ξ_KL_* and the K-fluorescent yields *ω_Kα_* and *ω_Kβ_* which express the probability of K-characteristic radiation of *Kα* and *Kβ* photons, as given below:

(6) $$if\;\left\{\begin{array}{l} R_{4}\leq \xi_{KL}\;and\;R_{5}\leq \omega_{K\alpha },\;then\;K_{\alpha }\;photons\;are\;produced\\ \xi_{KL}<R_{4}\leq 1\;and\;R_{6}\leq \omega_{K\beta },\;then\;K_{\beta }\;photons\;are\;produced \end{array}\right.$$

where *R*_4_, *R*_5_ and *R*_6_ are random numbers, uniformly distributed in the interval (0, 1]. All required parameters for iodine element are summarized in Table 1 [21,22].

Just after their production, the history of K-photons was considered to start with initial direction angles (polar and azimuthal) determined by an isotropic distribution and initially follows a step length *KS*_1_ which was obtained as follows:

(7) $$KS_{1}=-\frac{1}{{\left(\mu_{att}\right)}_{Tissue}(E)}$$

where *R*_7_ is a random number uniformly distributed in the interval (0, 1] and (*μ_att_*)*_Tissue_*(*E*) is the linear attenuation coefficient of normal tissue (fibrous) or tumor according to the tissue under investigation. The effect of iodine on tissue attenuation was not taken into account. Thereafter, the co-ordinates of the K-photon site were calculated as given below:

(8) $$\left(\begin{array}{l} x_{n+1}\\ y_{n+1}\\ z_{n+1} \end{array}\right)=\left(\begin{array}{l} x_{n}\\ y_{n}\\ z_{n} \end{array}\right)+KS_{1}\left(\begin{array}{l} a\\ b\\ c \end{array}\right)$$

where (*x_n_*, *y_n_*, *z_n_*) and (*x_n_*_+1_, *y_n_*_+1_, *z_n_*_+1_) are the coordinates of two successive sites, the primary and the secondary, and (*α*, *b*, *c*) is a vector representing the direction cosines of the K-photon trajectory, given by (*α*, *b*, *c*) = (*sinθcosφ*, *sinθsinφ*, *cosθ*), where *θ* and *φ* are the polar and azimuthal angles, respectively, which were initially determined from the isotropic distribution. Thereafter, if *z_n_*_+1_ − *z_n_* > *d*_2_ − *S*_2_′, the K photon history continues in the third layer traveling the remaining path length *KS*_2_ = *KS*_1_ − ((*d*_2_ − *S*_2_′)/*cosθ*) which is converted for the new tissue, as given below:

(9) $$KS_{2}\prime =KS_{2}\frac{{\left(\mu_{att}\right)}_{Tissue}(E)}{{\left(\mu_{att}\right)}_{Fat}(E)}$$

Thereafter, the co-ordinates of the K-photon site were calculated again as given below:

(10) $$\left(\begin{array}{l} x_{n+2}\\ y_{n+2}\\ z_{n+2} \end{array}\right)=\left(\begin{array}{l} x_{n+1}\\ y_{n+1}\\ z_{n+1} \end{array}\right)+KS_{2}\prime \left(\begin{array}{l} a\\ b\\ c \end{array}\right)$$

where (*x_n_*_+2_, *y_n_*_+2_, *z_n_*_+2_) are the final coordinates of the K-photon. Finally, the history of the K-photon was considered to terminate as soon as the following conditions are met with respect to the dimensions of the initial breast geometry, as given below:

(11) $$if\;\left\{\begin{array}{l} 0<x_{n+2}\leq x_{\dim ension}\\ 0<y_{n+2}\leq y_{\dim ension}\\ z_{n+2}>z_{\dim ension}\;\;\;\;\;\;\;\;\; \end{array}\right.$$

**Figure 4:**
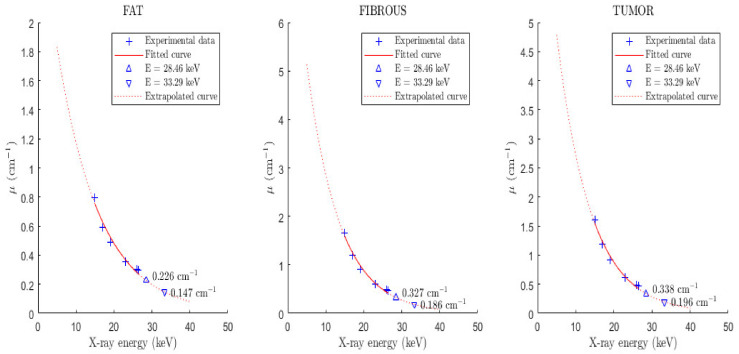
Figure illustrates the exponential fitting for the extraction of tissue (fat, fibrous, tumor) linear attenuation coefficients at X-ray energies 11.20 keV, 12.50 keV, 28.46 keV and 33.29 keV, respectively. The experimental data were taken from Chen et al. [23].

The attenuation properties of the tissues were based on published experimental measurements of the linear attenuation coefficients of fat, fibrous and tumor tissues [23]. Based on these values, exponential fitting was carried out in order to extract values at lower energies and specifically the linear attenuation coefficients at 28.46 keV and 33.29 keV, respectively, as shown in Figure 4.

Thereafter, the emitted K-photon impinges on an *Si* detector and follows a step length *KS_Detector_* until interaction, which is given below:

(12) $$KS_{Detector}=-\frac{1}{{\left(\mu_{att}\right)}_{Detector}(E)}\ln R_{8}$$

where *R*_8_ is a random number uniformly distributed in the interval (0, 1], (*μ_att_*)*_Detector_*(*E*) is the linear attenuation coefficient of the *Si* detector (with density 2.33 g/cm^3^). If *KS_Detector_* ≤ *Th*, where *Th* is the detector thickness, the K-photon is assumed to interact. In the present study, a 700 µm converter layer thickness was considered, which is encountered in Medipix2 readout system assembled into a full mammography system [24]. Only those K-photons that undergo photoelectric absorption were considered the final K-photons counted by the detector. Setting a random number *R*_9_, uniformly distributed in the interval (0, 1], the K-photon is assumed to undergo photoelectric absorption if *R*_9_ ≤ (*p_pe_*)*_Detector_*(*E*), as given below:

(13) $${\left(p_{pe}\right)}_{Detector}(E)=\frac{{\left(\mu_{pe}\right)}_{Detector}(E)}{{\left(\mu_{att}\right)}_{Detector}(E)}$$

where (*μ_pe_*)*_Detector_*(*E*) is the linear coefficient of photoelectric effect and (*μ_att_*)*_Detector_*(*E*) is the linear coefficient of the total attenuation regarding the *Si* detector at the corresponding energy *E*. The distance between the sample and the detector was not taken into account in the present study.

Overall, the simulation of the initial incident X-ray beam was assumed to terminate (the termination of the incident X-ray photons histories upon the breast) when the Entrance Surface Air Kerma (ESAK) was evaluated to be 10 mGy and 15 mGy, which are representative ESAK reference values for breast thicknesses of 4 cm and 8 cm, respectively [25,26]. The ESAK (μGy) was calculated using the following relationship [27,28]:

(14) $$ESAK=\sum_{E_{i}=\min}^{E_{i}=\max}\left(1.83\times 10^{-3}\;\Phi_{0}\left({Ε}_{i}\right)\;{Ε}_{i}{\left(\mu_{en}\left(E_{i}\right)/\rho \right)}_{air}/115\right)$$

where *Φ_0_*(*E_i_*) is the X-ray spectrum (Mo/Mo, W/Rh) given in photons/mm^2^ at energy *E_i_* and (*µ_en_*(*E_i_*)/*ρ*)*_air_* is the X-ray mass absorption coefficient of air at energy *E_i_*.

## 3. Results

Table 2 presents the results regarding the number of K-X-rays (*K_α_* and *K_β_*_1_) produced and emitted for the geometry illustrated in Figure 3 and described in Section II.C. Results are provided for: (a) two mammographic spectra: (i) 40 kV- W/Al (1.6 mm) and 50 kV- W/Al (1.6 mm) with additional filtration of 0.3 mm Gd; (b) two types of tissue (fibrous and tumor) with thicknesses ranging from 0.2 mm up to 2.5 mm; and (c) external fat tissue of 4 cm thickness. The number of K-X-rays was evaluated considering the initial number of X-ray photons corresponding to 10 mGy ESAK (the accuracy derived from all the counted cases). This amount of incident X-rays changes according to the type of the X-ray spectrum and depends on the X-ray energy and the corresponding mass absorption coefficient of air at that energy, as described in Equation (14). Table 3 presents the results under similar conditions of X-ray spectra and internal tissue characteristics; however, the thickness of the external fat tissue thickness was considered 8 cm and the number of K-X-rays was evaluated considering the initial number of X-ray photons corresponding to 15 mGy ESAK. Numerical values are expressed by the mean value and their corresponding standard deviation.

The combination of the aforementioned factors determines the X-ray beam photon fluence which is found to yield particularly lower values examining the 40 kV X-ray spectrum (154,325,927 ± 2361 and 231,489,318 ± 1873 for 10 mGy and 15 mGy ESAK, respectively) compared to that of the 50 kV X-ray spectrum (208,131,504 ± 11,054 and 312,198,595 ± 11,587 for 10 mGy and 15 mGy ESAK, respectively), mainly due to the reduction in rates of air coefficients with energy. In addition, this difference was found to affect the numerical data regarding the production and emission of K-X-rays for all cases examined in the present study. Within the framework of Monte Carlo simulation modeling, the results showed the following: (a) the production of K-X-rays was considerably higher than the emission of K-X-rays due to the reabsorption within the tissue after production (either in the internal fibrous/tumor tissue or in the external fat tissue); (b) although their lower energy (K_α_: 28.46 keV and K_β1_: 33.29 keV), the number of K_α_ X-rays was found to be considerably higher than the number of K_β1_ X-rays due the high value of KL relaxation probability (0.820), which dominates the production of K_α_ X-rays; (c) the emission of K-X-rays increases with the corresponding increase in the internal tissue (fibrous or tumor) thickness.

**Figure 5:**
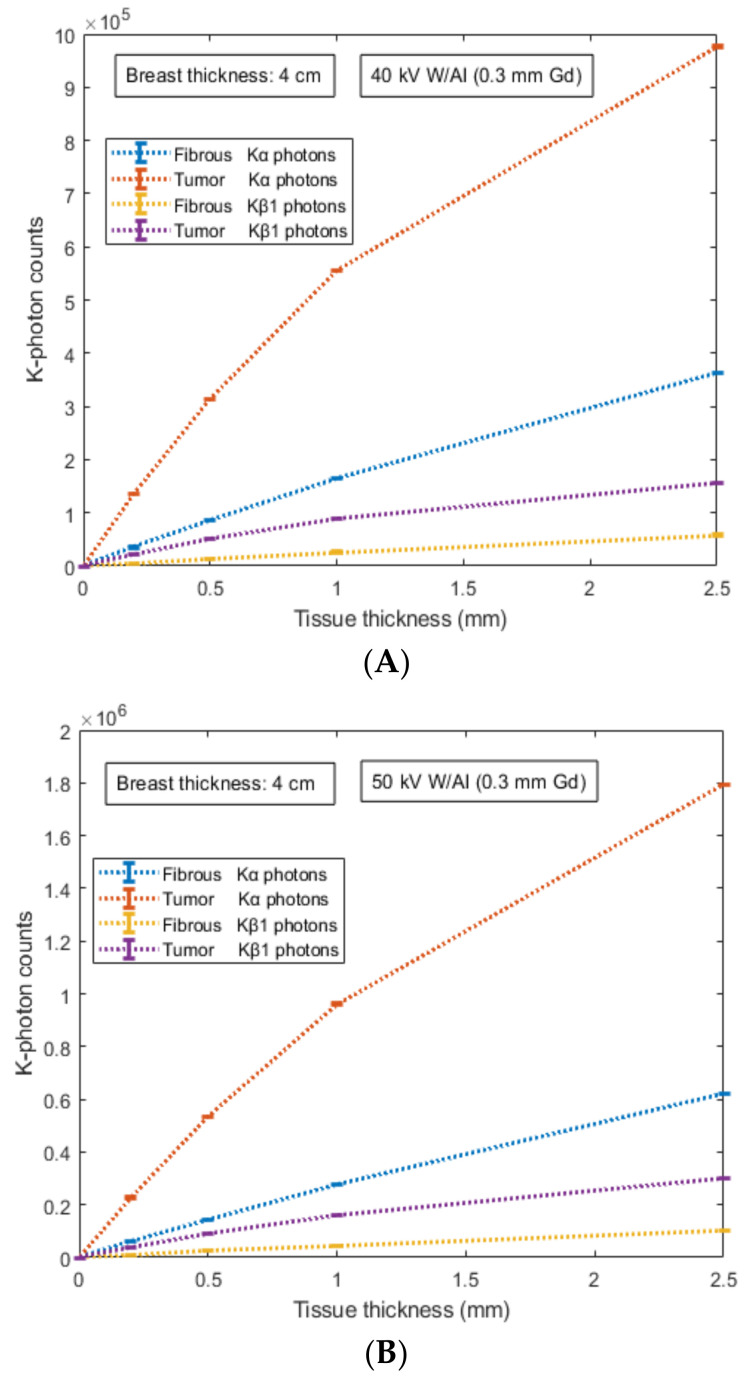
Figure shows the number of *K*-X-rays (*K_α_* and *K_β_*_1_) counted by the 700 µm converter layer of the Si detector. Results are provided for: (a) two X-ray spectra: (**A**) 40 kV- W/Al (1.6 mm) and (**B**) 50 kV- W/Al (1.6 mm) with additional filtration 0.3 mm Gd; (b) two types of internal tissue (fibrous and tumor) with thicknesses ranging from 0.2 mm up to 2.5 mm; and (c) external fat tissue of 4 cm thickness. Numerical values are expressed by the mean value and their corresponding standard deviation.

**Figure 6:**
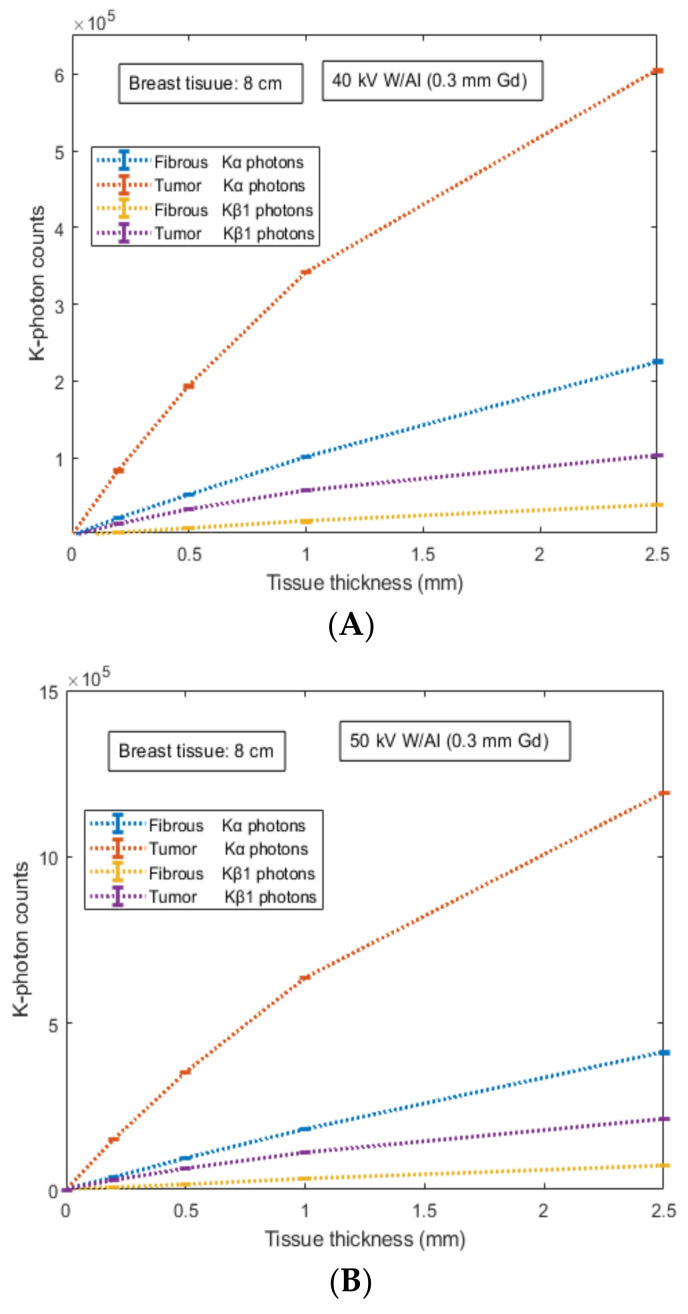
Figure shows the number of *K*-X-rays (*K_α_* and *K_β_*_1_) counted by the 700 µm converter layer of Si detector. Results are provided for: (a) two X-ray spectra: (**A**) 40 kV- W/Al (1.6 mm) and (**B**) 50 kV- W/Al (1.6 mm) with additional filtration 0.3 mm Gd; (b) two types of internal tissue (fibrous and tumor) with thicknesses ranging from 0.2 mm up to 2.5 mm; and (c) external fat tissue of 8 cm thickness. Numerical values are expressed by the mean value and their corresponding standard deviation.

The number of K-X-rays (*K_α_* and *K_β_*_1_) counted by the 700 µm converter layer of the Si detector is shown in Figure 5 and Figure 6 for external fat tissue of 4 cm and 8 cm, respectively. Results are provided for both mammographic spectra and the two types of internal tissue (fibrous and tumor) with thicknesses ranging from 0.2 mm up to 2.5 mm. Results showed that the number of counted K-X-rays was found higher for the 50 kV X-ray spectrum compared to that of the 40 kV. For example, for tissue thickness of 1 mm, the number of counted K-X-rays was found to be: (i) 135,447 ± 251 (tumor) and 35,027 ± 134 (fibrous) for the 40 kV case and 227,123 ± 389 (tumor) and 58,557 ± 239 (fibrous) for the 50 kV case.

Data correspond to 67.7% (tumor) and 67.2% (fibrous) increase for Κ_α_ counted photons and 4 cm external fat tissue, (ii) 69,834 ± 202 (tumor) and 17,999 ± 145 (fibrous) for the 40 kV case and 148,876 ± 167 (tumor) and 38,330 ± 124 (fibrous) for the 50 kV case. Data correspond to 113% for both tissues (tumor and fibrous) increase for Κ_α_ counted photons and 8 cm external fat tissue. Thus, the 50 kV X-ray spectrum and the case of Κ_α_-photons provide a quantitative advantage over other conditions. This is mainly due to the higher available X-rays above the K-edge in the energy interval 40–50 keV.

In order to provide a qualitative analysis of the results, the ratio of counted *K*-X-rays between tumor and fibrous tissue was evaluated, as provided in Table 4. Comparing the two X-ray spectra as well as the type of *K*-X-rays (i.e., *Κ_α_* and *K_β_*_1_), there is no qualitative difference between the tumor and the fibrous tissue considering the two cases of external fat tissue, 4 cm and 8 cm (i.e., their ratio was found to be almost similar). However, a point worth commenting on is that the tissue abnormality indication is more obvious for internal tissues of lower thickness. For instance, the ratio is approximately 3.87 for internal tissue thicknesses of 0.2 mm and thereafter decreases down to approximately 2.68 for internal tissue thicknesses of 2.5 mm, regarding the case of 40 kV. Another important note is that the ratio was found to be higher at 50 kV than at 40 kV (a higher difference was observed at 2.50 mm). The aforementioned ratio shows that the photon-counting mode of *K*-photons can complement the energy integrating mode and act as an additional diagnostic tool of functional features during an X-ray irradiative examination.

## 4. Discussion

The present manuscript introduces a possible indication of breast tissue abnormality by employing a hybrid detective system for image acquisition (with morphological and functional features). The manuscript presents the main idea, introduces the proof of concept of the basic components, and attempts to address some important issues; however, it is impossible to cover all possible uncertainties and limitations embedded in the proposed structure. Several issues need further examination, discussion, and future clinical prospects in order to connect the theoretical considerations with medical practice. From the point of view of research and development, special attention should be also given to different perspectives [29,30,31]: (a) the contribution of image formation in diagnosis and (b) the scientific principles of medical image science [32]. In other words, novel techniques should be based on the observer assessment of specific patient examinations (e.g., the perception of the medical doctor for valid decision) and on the physical and technological parameters that influence the optimization of the medical image which thereafter contributes to early detection of pathological features [33].

One important issue which is related to the described instrumentation scheme is the utilization of a hybrid detective system. This system is referred to as having the capability of processing the absorbed X-ray beam either by integrating the energy of the X-ray photons or by counting each X-ray photon separately. In recent years, new innovative detective systems, called Medipix and Timepix, have been introduced into the field of medical imaging transferring the knowledge gained from high-energy physics at CERN. These types of sensor readout chips satisfy the aforementioned demands for both micro-calcifications detection (a crucial parameter in mammography examinations) accompanied by full spectroscopic X-ray imaging [24,34,35]. A significant aspect of the combined configuration is also the ability to allow the existence of more than one contrast agent since their separation (i.e., the measurement of multiple X-ray energy profiles) is enabled through the multiple thresholds of recently developed energy-resolved photon-counting detectors [36,37]. In addition, such detectors can employ dual energy techniques for different tissue contrast enhancement or three-dimensional structures that may reduce the level of noise by minimizing the charge sharing between adjacent pixels occurring in standard planar devices [38].

Another factor that may play a significant role in the qualitative accuracy of the examination is the assessment of the contrast agent concentration with respect to the examination time. Special treatment is required to determine: (a) the optimum time period in order to proceed to the X-ray irradiation or (b) the appropriate time frames in case of multiple X-ray exposures. In any case, it is imperative for anybody administering contrast agents to be intimately familiar with their characteristics, indications as well as their potential side effects (e.g., the ability to recognize adverse reactions promptly and treat them effectively and rapidly). This feature of transport and binding procedures is important since it may be responsible for the variation of contrast agent concentration and may become a crucial factor for characterizing abnormality. Recent studies on nanoparticle development [39] could also create a bust in contrast agent research for such applications.

Another issue is the compression of the breast. In breast imaging, breast compression (mainly in X-ray projection) affects the fraction of the transmitted radiation and spreads the dense tissue of the breast indicating abnormalities that might be hidden. However, breast compression reduces the blood volume which plays a crucial role in tissue contrast enhancement for the characterization of pathological features. On the other hand, avoiding compression is a more suitable arrangement for 3D imaging [10]. A point worth mentioning is that in recent studies, the reduction of breast compression in tomosynthesis without a negative cause on radiation dose or image quality was suggested [40]. Therefore, the question remains for the present proposal. Breast compression, how much is enough? [41].

## 5. Conclusions

The present manuscript attempts to present a proof of concept from a physics perspective of a hybrid detective system based on the utilization of contrast agents within the framework of breast imaging screening. Based on the Monte Carlo modeling, the basic findings are summarized below: (a) numerical evaluations showed the possible contribution of *K*-X-ray counting of a contrast agent during an X-ray irradiative examination; (b) there was a quantitative advantage of using X-ray spectra of 50 kV W/Al and by counting the *Κ_α_* photons; however, the qualitative difference on the tumor–fibrous ratio is more obvious at lower tissue thickness values; (c) the amount of *K_β_*_1_ photons provides the capability to play a complementary role on fibrous/tumor characterization; however, they would be more beneficial in higher tissue thickness values. In conclusion, by utilizing suitable contrast agents as ‘‘fingerprint’’ tissue abnormalities, the acquisition of combined morphological and functional imaging features (through the counting of *K*-X-rays) could enhance breast imaging in its present form and lead to advanced prognostic capabilities of breast cancer.

## Figures and Tables

**Table 1:** Physical parameters and coefficients of iodine element.

	Contrast Agent-Iodine (I)
K-edge (keV)	33.18
Fluorescent yield *ω_Kα_*	0.841 ^a^
X-ray energy (keV) *Κα*	28.46
Fluorescent yield *ω_Kβ_*	0.900 ^b^
X-ray energy (keV) *Κβ*1	33.29
Probability of *KL* relaxation *ξ_KL_*	0.820 ^c^

^a^ taken from Hubbell et al. [22], ^b^ consideration of the present study, ^c^ taken from Cullen et al. [21].

**Table 2:** Table shows the number of Iodine *K*-X-rays (*K_α_* and *K_β_*_1_) produced and emitted as well as the initial number of X-ray photons (corresponding to 10 mGy ESAK). Results are provided for: (a) two X-ray spectra: 40 kV- W/Al (1.6 mm) and 50 kV- W/Al (1.6 mm) with additional filtration 0.3 mm Gd; (b) two types of internal tissue (fibrous and tumor) with thicknesses ranging from 0.2 mm up to 2.5 mm; and (c) external fat tissue of 4 cm thickness. Numerical values are expressed by the mean value and their corresponding standard deviation.

	Κα Produced	Κα Emitted	Κβ1 Produced	Κβ1 Emitted
Tissue thickness (mm)	40 kV W/Al (0.3 mm Gd)			
	ESAK 10 mGy: 154,325,927 ± 2361 X-ray photons			
	**Fibrous tissue**			
**0.20**	919,530 ± 1113	180,213 ± 91	216,270 ± 585	46,657 ± 134
**0.50**	2,259,247 ± 303	439,864 ± 318	531,387 ± 314	113,560 ± 243
**1.00**	4,390,417 ± 1123	845,360 ± 1441	1,031,301 ± 689	218,528 ± 210
**2.50**	10,069,414 ± 6029	1,871,363 ± 662	2,364,725 ± 577	485,761 ± 558
	**Tumor tissue**			
**0.20**	3,554,987 ± 1547	696,311 ± 525	834,560 ± 1110	179,404 ± 303
**0.50**	8,288,675 ± 1295	1,613,178 ± 176	1,946,062 ± 160	416,505 ± 316
**1.00**	14,825,168 ± 5152	2,847,935 ± 487	3,480,900 ± 1381	735,349 ± 988
**2.50**	27,282,049 ± 8256	5,018,295 ± 1782	6,411,497 ± 3725	1,304,219 ± 1618
	50 kV W/Al (0.3 mm Gd)			
	ESAK 10 mGy: 208,131,504 ± 11,054 X-ray photons			
	**Fibrous tissue**			
**0.2** **0**	1,528,085 ± 950	299,099 ± 672	359,320 ± 227	77,464 ± 362
**0.50**	3,769,500 ± 1747	733,758 ± 1030	886,339 ± 886	189,812 ± 236
**1.00**	7,359,554 ± 3712	1,414,941 ± 742	1,729,163 ± 243	366,219 ± 397
**2.50**	17,167,289 ± 2508	3,193,661 ± 1358	4,030,857 ± 878	829,263 ± 962
	**Tumor tissue**			
**0.20**	5,946,659 ± 302	1,164,779 ± 1396	1,397,721 ± 558	300,511 ± 824
**0.50**	14,048,481 ± 3716	2,734,730 ± 1064	3,298,319 ± 1223	704,555 ± 647
**1.00**	25,675,571 ± 3529	4,940,486 ± 3296	6,028,654 ± 1281	1,275,854 ± 751
**2.50**	49,935,784 ± 3291	9,208,489 ± 3261	11,721,499 ± 2947	2,390,102 ± 1366

**Table 3:** Table shows the number of Iodine *K*-X-rays (*K_α_* and *K_β_*_1_) produced and emitted as well as the initial number of X-ray photons (corresponding to 15 mGy ESAK). Results are provided for: (a) two X-ray spectra: 40 kV- W/Al (1.6 mm) and 50 kV- W/Al (1.6 mm) with additional filtration 0.3 mm Gd; (b) two types of internal tissue (fibrous and tumor) with thicknesses ranging from 0.2 mm up to 2.5 mm; and (c) external fat tissue of 8 cm thickness. Numerical values are expressed by the mean value and their corresponding standard deviation.

	Κα Produced	Κα Emitted	Κβ1 Produced	Κβ1 Emitted
Tissue thickness (mm)	40 kV W/Al (0.3 mm Gd)			
	ESAK 15 mGy: 231,489,318 ± 1873 X-ray photons			
	**Fibrous tissue**			
**0.20**	1,086,583 ± 693	111,018 ± 172	255,284 ± 856	30,692 ± 133
**0.50**	2,668,106 ± 128	271,053 ± 131	626,624 ± 804	74,837 ± 277
**1.00**	5,182,404 ± 1631	521,206 ± 614	1,216,283 ± 1555	144,092 ± 164
**2.50**	11,883,820 ± 1862	1,158,467 ± 891	2,792,623 ± 849	322,277 ± 618
	**Tumor tissue**			
**0.20**	4,195,170 ± 458	429,199 ± 132	985,172 ± 553	118,587 ± 355
**0.50**	9,782,535 ± 3045	995,528 ± 776	2,297,811 ± 1913	274,695 ± 243
**1.00**	17,496,435 ± 4343	1,758,550 ± 2252	4,112,562 ± 474	486,680 ± 484
**2.50**	32,188,347 ± 913	3,107,396 ± 1886	7,563,711 ± 3166	864,557 ± 604
	50 kV W/Al (Gd filtration)			
	ESAK 15 mGy: 312,198,595 ± 11,587 X-ray photons			
	**Fibrous tissue**			
**0.2** **0**	1,927,864 ± 480	196,690 ± 354	452,256 ± 791	54,170 ± 116
**0.50**	4,753,395 ± 2088	482,327 ± 408	1,115,946 ± 404	133,064 ± 253
**1.00**	9,279,887 ± 1131	932,832 ± 612	2,180,559 ± 511	258,119 ± 317
**2.50**	21,657,692 ± 2070	2,112,970 ± 1711	5,086,165 ± 1837	586,491 ± 1310
	**Tumor tissue**			
**0.20**	7,494,827 ± 1304	765,266 ± 340	1,761,087 ± 1369	211,620 ± 758
**0.50**	17,722,602 ± 6765	1,801,619 ± 619	4,165,397 ± 1660	497,862 ± 461
**1.00**	32,418,446 ± 9013	3,261,592 ± 1376	7,612,154 ± 2321	901,771 ± 1504
**2.50**	63,219,666 ± 6066	6,123,979 ± 417	14,856,997 ± 3051	1,704,321 ± 1617

**Table 4:** Table shows the ratio of *K*-X-rays counted (*K_α_* and *K_β_*_1_) by the 700 µm Si converter layer between tumor and fibrous tissue. Results are provided for: (a) two X-ray spectra: 40 kV- W/Al (1.6 mm) and 50 kV- W/Al (1.6 mm) with additional filtration 0.3 mm Gd; (b) two types of internal tissue (fibrous and tumor) with thicknesses ranging from 0.2 mm up to 2.5 mm; and (c) external fat tissue of 4 cm and 8 cm thickness. Numerical evaluations obtained by the deviation of their mean value.

	Ratio of *Κα* Counted	Ratio of *Κβ*1 Counted	Ratio of *Κα* Counted	Ratio of *Κβ*1 Counted
	40 kV W/Al (0.3 mm Gd)			
Tissue thickness(mm)	Fat tissue: 4 cm		Fat tissue: 8 cm	
**0.20**	3.87	3.88	3.86	3.86
**0.50**	3.67	3.68	3.66	3.66
**1.00**	3.37	3.35	3.38	3.38
**2.50**	2.68	2.68	2.68	2.68
	**50 kV W/Al (0.3** **mm Gd)**			
	**Fat tissue: 4 cm**		**Fat tissue: 8 cm**	
**0.20**	3.88	3.85	3.88	3.87
**0.50**	3.72	3.72	3.74	3.75
**1.00**	3.49	3.48	3.50	3.48
**2.50**	2.89	2.89	2.90	2.90

## Data Availability

Data are contained within the article.
